# Correction to “Odd–Even Effect in Photoreactivity and Transistor Property of Benzo[*b*]‑benzo[4,5]thieno[2,3‑*d*]thiophene Tethered with a Diacetylenic Group through a Spacer Chain”

**DOI:** 10.1021/acs.chemmater.6c00675

**Published:** 2026-04-14

**Authors:** Samala Venkateswarlu, Fang-Ju Lin, Yu-Tai Tao

The mobility values reported for compound **15** in the original manuscript were incorrect due to a data-processing oversight. In the first paragraph of the “Device Performance” section of the Results and Discussions, the second and third sentences with the corrected description should read as follows:

“Higher mobilities were recorded for the irradiated films of **15** and **17**, with mobilities of 0.13 and 0.26 cm^2^ V^–1^ s^–1^, respectively. In contrast, devices fabricated from irradiated films of BTBT-diacetylenes **14** and **16** exhibited significantly lower mobilities of 0.058 and 0.075 cm^2^ V^–1^ s^–1^.

Only the corrected data for compound **15** in Table 2 are shown below:

**Table 2:** Crystallographic Parameters and Bottom-Gate Top-Contact (BGTC) OTFT Device Performance for Sheared Films

compd	condition	*d*_(001)_ (Å)	*d*_(020)_ (Å)	*d*_(020)′_ (Å)	μ_h,adv_ (μ_h,max_) (cm^2^ V^–1^ s^–1^)^a^	*V*_th_ (V)	*I*_on_/*I*_off_
15	unirradiated film	59.58	4.27		0.048 ± 0.012 (0.066)	–35 to −55	10^5^–10^7^

a Average mobilities and maximum values of hole mobility are shown in parentheses (more than 20 devices were tested for each condition).

